# Acid scarification as a potent treatment for an in vitro germination of mature endozoochorous *Vanilla planifolia* seeds

**DOI:** 10.1186/s40529-023-00374-z

**Published:** 2023-04-17

**Authors:** Jan Šoch, Josef Šonka, Jan Ponert

**Affiliations:** 1 Department of Experimental Plant Biology, Faculty of Science, Charles University, Viničná 5, 128 44 Prague, Czech Republic; 2 Prague Botanical Garden, Trojská 800/196, 171 00 Prague, Czech Republic; 3 Institute of Botany, Czech Academy of Sciences, 252 43 Průhonice, Czech Republic

**Keywords:** Seed germination, *Vanilla planifolia*, Acid scarification, Orchid, In vitro cultivation, Hydrochloric acid, HCl, Calcium hypochlorite, Sulfuric acid

## Abstract

**Background:**

*Vanilla planifolia* is the most widely cultivated species of vanilla with high economic importance. However, seed germination under artificial conditions is difficult and yields low germination percentages. The seeds are adapted to endozoochorous dispersal, and we therefore tried to simulate the conditions in the digestive tract by acid scarification of seeds.

**Results:**

Immature seeds lacking dormancy, used as a control, showed the highest germination percentage. Among the treatments tested for mature seeds, the hydrochloric acid treatments were significantly the best in breaking dormancy and inducing germination, irrespective of the acid concentration and the presence of pepsin. Conventional treatment with a hypochlorite solution induced much lower germination percentage. Sulphuric acid at concentration 50% was too strong and caused damage to the seeds. Important factor is also high cultivation temperature 30 °C as there was nearly no germination at 25 °C.

**Conclusions:**

Our protocol significantly improves the efficiency of generative propagation of vanilla and allows for significantly higher germination percentages than previously described. The strongly positive effect of hydrochloric acid may be related to the adaptation of seeds to endozoochorous dispersal.

**Supplementary Information:**

The online version contains supplementary material available at 10.1186/s40529-023-00374-z.

## Backgound

Vanilla (mostly *Vanilla planifolia*) is one of the most expensive spices in the world (Parthasarathy et al. 2008) and its global market size is expected to be about 950 million USD by 2024 with expected compound annual growth rate (CAGR) of 13% (MRFR 2019). Despite this huge demand on vanilla production, its artificial propagation is mostly limited to vegetative propagation and growing from seeds is still highly challenging and unpredictable. *Vanilla* belongs to the family Orchidaceae and all orchids produce tiny “dust seeds” with undifferentiated embryo, no endosperm and very little reserves (Arditti and Ghani 2000; Kristiansen et al. 2001). After germination, embryo grows out of the testa into a larger structure called protocorm (Bernard 1909; Rasmussen 1990; Yeung 2017). Considerably later, the first meristem is formed on protocorm and the orchid shoot starts to grow (Yeung et al. 2018). In nature, young orchids depend on nutrition provided by symbiotic mycorrhizal fungi (Selosse et al. 2016; Tĕšitel et al. 2018). Under artificial conditions, it can be difficult to germinate orchids with their symbiotic fungi, especially because of great fungal diversity and frequent specificity between mycorrhizal partners (Dearnaley 2007; Jacquemyn et al. 2017). The fungus is therefore commonly substituted by a nutrient solution and the orchid sowings are performed under aseptic in vitro conditions (Arditti 1967; Rasmussen 1995; Yam and Arditti 2017).

However, optimizing the nutrient solution (cultivation medium) is not the only task which needs to be addressed. Orchid seeds frequently exhibit some kind of dormancy which needs to be broken artificially (see Rasmussen 1995 and references therein). Majority of orchid seeds do not germinate after a contact with water alone and they need a treatment with a corrosive or alkaline solution to induce germination. Most widely used are calcium- or natrium- hypochlorite solutions, usually with an addition of some wetting agent (see Rasmussen 1995; Yam and Arditti 2017; Yeung et al. 2018). It is sometimes combined with a pre-treatment with 50–96% ethanol (e.g., Vejsadová 2006; Ponert et al. 2013, 2021). Such a treatment efficiently induces germination of many orchid species, but others exhibit more complex and diverse requirements. Some of these reluctant orchid species have been successfully germinated after the abovementioned hypochlorite treatment combined with (i) a pre-treatment with 2% sulphuric acid (e.g. Malmgren 1993; Ponert et al. 2013; Malmgren and Nyström 2020), (ii) cold stratification (e.g. Ballard 1987; Coke 1990; Rasmussen 1992; Ponert et al. 2013; Malmgren and Nyström 2020), or incubation at specific temperature (Nakamura 1982; Johnson and Kane 2012). The positive﻿ effect of bleaching in hypochlorite solutions, soaking in ethanol and weak acid could be attributed mainly to a degradation of the seed coat (e.g., Lee et al. 2007; Barsberg et al. 2013; Magrini et al. 2019; Pierce et al. 2019). The hypochlorite solutions exhibit a high pH and strong oxidative effects on a wide range of compounds including polymers of plant cell walls (Arditti 1967; Rasmussen 1995; Zeng et al. 2014). Ethanol is a good dissolvent of waxes and some other smaller hydrophobic molecules (Holser 2009). Taking together, requirements of different orchid species are highly diverse. Generally, two main steps can be necessary to deal with: (i) penetration of the seed testa in species where the testa is impermeable, and (ii) stimulation of germination by chemical compounds or temperature regime in species which do not germinate even when water enters the seed.

The cultivation procedures summarized above have been optimized for a vast number of orchids. However, some others still resist to our attempts. One of such difficult orchids is the genus *Vanilla*, including horticulturally important species like *V. planifolia*. This genus is quite different from other artificially cultivated orchids in its evolutionary isolation (it is a member of a relatively small subfamily Vanilloideae) and in its endozoochorous seed dispersal (Soto Arenas and Dressler 2013; Pansarin 2021; Pansarin and Suetsugu 2022). Seeds of a vast majority of orchids (and of all other cultivated species) are anemochorous, elongated, and light-coloured (Arditti and Ghani 2000). The seeds of *Vanilla* possess black glossy coat and they are globular (a common feature of endozoochorous seeds to reduce surface to volume ratio; Pakeman et al. 2002; Razanamandranto et al. 2004; Yeh et al. 2021).

As common in plant seeds, the impregnation of testa leading to its impermeability and the deposition of inhibitory substances into seeds take place late during the orchid seed development (Yamazaki and Miyoshi 2006; Zhang et al. 2013). Consequently, the reluctant orchid species which do not germinate after conventional treatments can be germinated from immature seeds with high germination rates (e.g. Yamazaki and Miyoshi 2006; Sgarbi et al. 2009; Pierce and Cerabolini 2011; Zhang et al. 2013). However, the proper time frame during the seed development which allows efficient germination can be very short and differs between species. This approach has been used for in vitro germination of *V. planifolia* (Yeh et al. 2021) and *V. siamensis* (Chaipanich et al. 2020) reaching relatively low germination percentages around 10%, likely missing the proper time frame. Another disadvantage of this approach is that the immature seeds cannot be easily stored and must be sown immediately (Rasmussen 1995). The establishment of protocol for efficient in vitro sowing of mature seeds is therefore of importance.

Several attempts have been made to germinate mature seeds of different *Vanilla* species in vitro, but with limited success. By far the highest germination percentages were achieved with *V. rivasii*, both axenically and symbiotically, which is probably specific to this species (Alomía et al. 2017). The highest germination percentages achieved in vitro with commercially important species reached around 30% and were achieved by two different approaches, both asymbiotically. Knudson (1950) disinfected seeds of *V. fragrans* with calcium hypochlorite and he transferred sowings to incubator with temperature 32 °C after long-time unsuccessful cultivation at regular temperature. Pansarin (2021) disinfected seeds of five species including *V. planifolia* with weaker sodium hypochlorite solution, but before that he briefly scarified the seeds with sulphuric acid (unspecified concentration for 60 s). Disinfection of seeds of *V. planifolia* with only sodium hypochlorite solution yielded much lower germination percentages (Pansarin 2021; Yeh et al. 2021). Symbiotic cultivations did not work well with any other species tested than the abovementioned *V. rivasii* (Alomía et al. 2017), including the commercially important *V. planifolia* and *V. odorata* (Porras-Alfaro and Bayman 2007; Alomía et al. 2017).

Germination of endozoochorous non-orchid seeds have been induced by mechanical scarification (Peco et al. 2006; Gunes et al. 2013; Kleyheeg et al. 2018), acid treatment with sulphuric acid (Gunes et al. 2013; Vazačová and Münzbergová 2013) or hydrochloric acid (Jaganathan et al. 2019) and by a treatment with pepsin in HCl solution (Pérez et al. 2005; Peco et al. 2006; Venier et al. 2012; Kleyheeg et al. 2018). However, most of these methods have not been tested in orchids. We therefore sought to develop a treatment allowing an efficient germination of the commercially important *Vanilla planifolia* seeds. We hypothesized, that the simulation of an acid environment of animal digestive system may induce *Vanilla* germination.

## Methods

### Seed collection

The plants of *Vanilla planifolia* Andrews were cultivated in a warm greenhouse (Department of Experimental Plant Biology, Faculty of Science, Charles University, Prague) with automatic ventilation and heating set to maintain minimum temperature 21 °C without artificial shading. Maximum summer temperature does not excess 38 °C and relative air humidity ranges from 50 to 99%. Fully open flowers were hand pollinated. To get ripe seeds, ripe fruits were collected when started to open (approximately 12 months after pollination, Additional file 1: Figure S1). The fruits were stored at + 25 °C in the dark and dry conditions for maximum 3 months. When the fruits dehisced and became brownish and dry, seeds were manually extracted. The immature seeds were collected at 10 months after pollination.

### Seed treatments

We tested treatments with hydrochloric and sulfuric acids which were used previously to induce germination of other endozoochorous seeds. We compared these experimental treatments with two types of controls: (i) immature seeds where a high germination percentage could be expected, and (ii) conventional treatments of ripe seeds with a chlorinated lime which were used previously with limited success (Knudson 1950). Complete experimental design is summarized in Table 1 and number of replications is given in Supplementary table 1.

**Table 1:** Disinfection and scarification treatment of vanilla seeds prior to sowing

Subject of disinfection	State of seeds	70% ethanol	Disinfectant	Time	Concentration	Tween-20	Pepsin	Sterile deionized water
Seeds	Mature	4 min	H_2_SO_4_	5 min	96%	No	No	3x
Seeds	Mature	4 min	H_2_SO_4_	15 min	96%	No	No	3x
Seeds	Mature	4 min	H_2_SO_4_	30 min	96%	No	No	3x
Seeds	Mature	4 min	HCl	30 min	0.1 M	Yes	Yes	3x
Seeds	mature	4 min	HCl	30 min	0.1 M	No	No	3x
Seeds	Mature	4 min	HCl	4 h	0.1 M	Yes	Yes	3x
Seeds	Mature	4 min	HCl	15 min	35 – 38%	No	No	3x
Seeds	Mature	4 min	H_2_SO_4_	3 min	50%	No	No	3x
Seeds	Mature	4 min	H_2_SO_4_	washed	50%	No	No	3x
Seeds	Mature	4 min	H_2_SO_4_	5 min	50%	No	No	3x
Seeds	Mature	4 min	Chlorinated lime	30 min	66 g/l	No	No	3x
Fruits	Immature	no	Chlorinated lime	5 min	20% w/v	Yes	No	1x

Disinfection and scarification treatment of *Vanilla planifolia* seeds prior to sowing. Tween-20 (1 drop per 200 ml) and pepsin (0.5 g/100 ml, porcine gastric mucosa, 250 U/mg, Sigma-Aldrich) were added into disinfection solution of selected treatments as indicated

Three immature vanilla fruits were surface disinfected by soaking in 20% w/v chlorinated lime (Kittfort Praha s.r.o.) with Tween-20 (1 drop per 200 ml) for 5 min and washed in sterile deionized water prior sowing. Seed pods were aseptically cut, and the seeds were transferred onto agar medium by sterile tools.

Mature seeds collected from dehisced vanilla fruits were treated with several oxidizing agents for disinfection and dormancy break prior to sowing. Treatment with chlorinated lime was performed using Luer plastic syringes as described in Ponert et al. (2011). Seeds were incubated for 4 min in 70% ethanol to remove sticky substance on their surface and then incubated for 30 min in chlorinated lime solution (66 g/l, as described in Knudson 1950) and then washed 3 times in sterile deionized water. Treatments with diluted hydrochloric acid were also performed in plastic syringes (Ponert et al. 2011), as weak solution of this acid did not dissolve nylon mesh. Seeds were incubated for 4 min in 70% ethanol and then incubated in 0.1 M HCl (Penta, Prague) solution for 30 min and in 0.1 M HCl solution with Tween-20 and pepsin (0.5 g/100 ml, porcine gastric mucosa, 250 U/mg, Sigma-Aldrich) for 30 min and 4 h; afterward the seeds were washed 3 times in sterile deionized water. All the abovementioned incubations were carried out at room temperature (22–27 °C).

Concentrated hydrochloric and sulfuric acid treatments had to be performed with sterile chemistry glassware and iron tools, because these acids dissolve nylon mesh. Seeds were transferred to a beaker and incubated for 4 min in 70% ethanol solution and then strained with colander and washed with tap water heavily. Later steps had to be conducted in laminar flowbox with sterile chemistry glassware and iron tools. The seeds were then transferred to a beaker with 50% H_2_SO_4_ (Penta, Prague) solution (seeds were either just washed or incubated for 5 min), or 96% H_2_SO_4_ (incubation for 5, 15 or 30 min) or 35–38% HCl (incubation for 15 min). The seeds were then strained with sterile iron colander (under which had to be placed rather large beaker, at least 0.3 l) and washed with sterile deionized water. Because dilution heat of both acids is rather huge (Sturtevant 1940; Kim and Roth 2001), seeds should be quickly washed with big excess of water to cool down (at least 0.2 l) and one should avoid inhaling released vapour. Washed seeds were aseptically transferred onto agar medium.

### In vitro cultivation conditions

Seeds were cultivated asymbiotically on BM1 medium (Himedia, cat.n. PT063; Additional file 2: Table S1) in plastic Petri dishes (9 cm) at 30 °C in dark for 5 months. We also tested different incubation temperature 25 °C, but only with seeds treated by the solution of chlorinated lime for 30 min. Petri dishes had to be double-sealed with a layer of parafilm (P-lab) and Leucopore tape (Duchefa) as a second layer to avoid melting and perforation of parafilm which could lead to a drying of medium and contaminations.

### Germination and growth analysis

Germination percentage of seeds was counted manually using ZEISS Stemi 305 Compact Greenough Stereo Microscope (magnification × 40). Germination percentage was computed as a ratio between seeds with ruptured and compact testa. Protocorms were photographed with Canon EOS 60D camera with Canon Macro EF 100 mm 1:2.8 l IS USM lens, and their size was measured in software ImageJ 1.50i as a maximum protocorm diameter following the procedure in Ponert and Lipavská (2017).

### Statistical analysis

Statistical analysis was performed using CRAN R 4.0.2 software (The R Core Team 2020) with package Rcmdr (Fox 2016). The normal distribution of data was tested with Shapiro–Wilk test (Shapiro and Wilk 1965), in case of ANOVA it was applied on standard residues of the model. Homoskedasticity of data was tested using Bartlett test (Bartlett 1937). Sample sizes are given in Additional file 3: Table S2. Data for testing differences among germination percentages were transformed with arcsin transformation ($x\prime = arcsin\sqrt x$)) to achieve normal distribution and tested with Welch F-test (Welch 1951), because the data were heteroscedastic. Data for testing differences among sizes of protocorms had hierarchical design and therefore they were tested with nested ANOVA with Petri dish as a random factor. Both Welch’s F-test and nested ANOVA were followed by Tukey HSD test (Tukey 1949).

## Results

### Germination

**Fig. 1:**
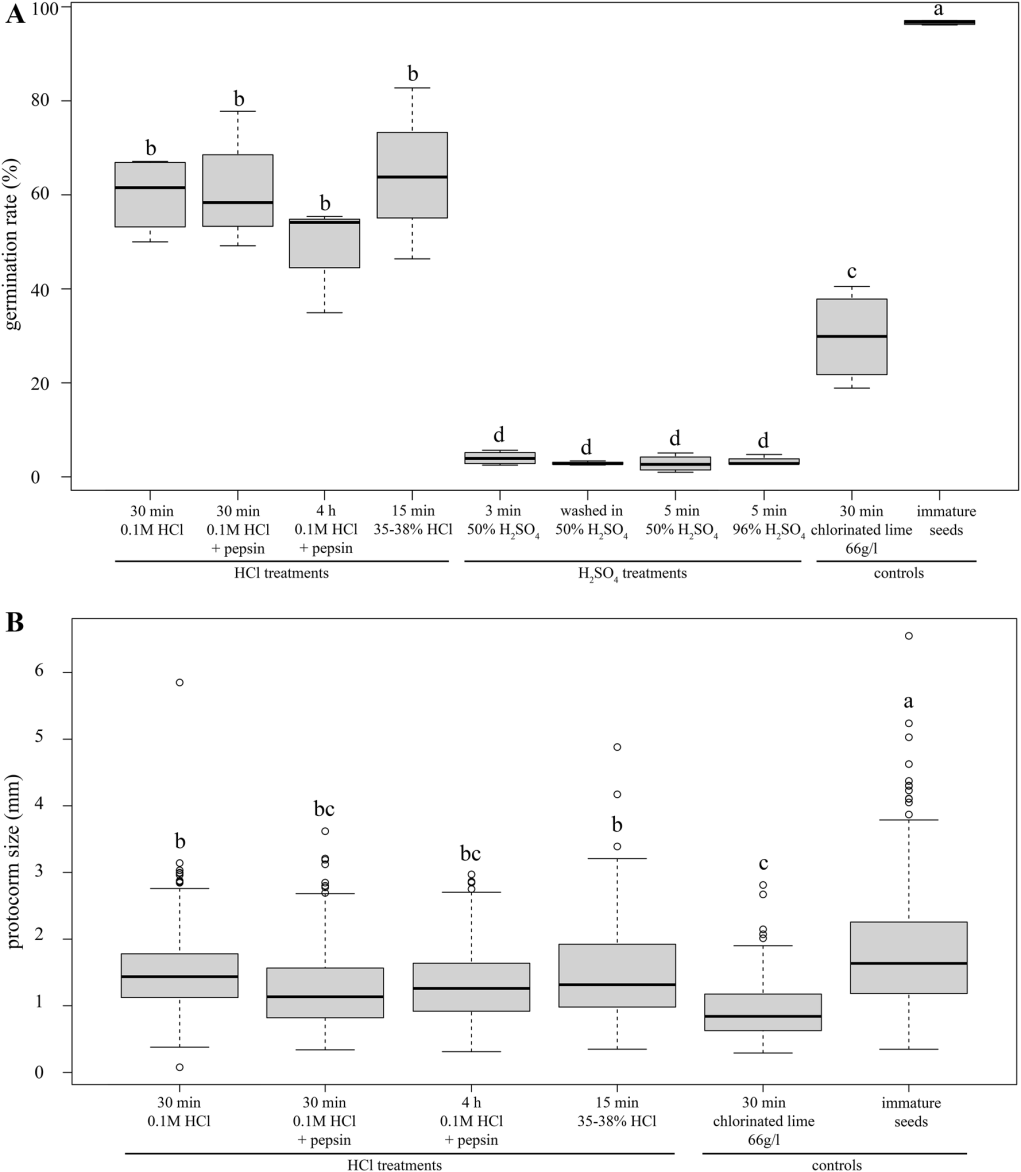
Germination percentage (**A**) and protocorm size (**B**) of *Vanilla planifolia* after different treatments of seeds measured 5 months after sowing. Immature seeds excerpted from surface sterilized unripe pods served as a positive control as they lack dormancy. Mature seeds treated with chlorinated lime represent conventional treatment published before. The cultures were incubated asymbiotically on medium BM1 at 30 °C in dark. Pepsin was used in concentration 0.5 g/100 ml. Different letters indicate significantly different groups of data according to the result of *post-hoc* multiple comparison test (α = 0.05). Boxplots compose of whiskers (minimum and maximum), edges of the box (lower and upper quartiles), dividing line (median) and points (outliers). Number of measured objects and other statistics can be found in Additional file 2: Table S1. Only those experimental variants that yielded a reasonable number of protocorms are included in (**B**)

Disinfection treatments had statistically very significant effect on the germination of vanilla seeds [F _(9,28)_ = 84.99, P < 2 × 10^–16^]. The difference was most pronounced in immature seeds, which germinated in nearly 100%, which is substantially more than any mature seeds regardless of their treatment (Fig. 1 A; Additional file 3: Table S2). It is also worth noting that variability in germination percentage of immature seeds was much smaller in comparison with germination percentage of all mature seed treatments. The hydrochloric acid treatments of vanilla mature seeds led to significantly highest germination percentage among mature seed treatments which reached approximately two thirds of germination percentage of immature seeds. However, the concentration of hydrochloric acid nor the presence of pepsin had a significant effect on the germination percentage of mature vanilla seeds. Significantly lower germination percentage was obtained by calcium hypochlorite treatment, which led to only approximately two thirds of germination percentage of hydrochloric acid treatments. Very low germination percentage was observed on all variants of sulfuric acid treatment of mature vanilla seeds. Two strongest sulfuric acid treatments (96% H_2_SO_4_ for 15 and 30 min) completely inhibited germination but even milder treatments (incubation in 96% H_2_SO_4_ for 5 min, wash in 50% H_2_SO_4_ and incubation in 50% H_2_SO_4_ for 5 min) led to significantly lowest germination percentages among all treatments, reaching only 3–4%. The seeds treated with H_2_SO_4_ were frequently disintegrated. We also observed effect of temperature with seeds tr﻿eated with solution of chlorinated lime for 30 min. No germination was observed at 25 °C whereas the germination percentage reached 29.7% (mean value; Fig. 1A) at 30 °C.

### Growth

**Fig. 2:**
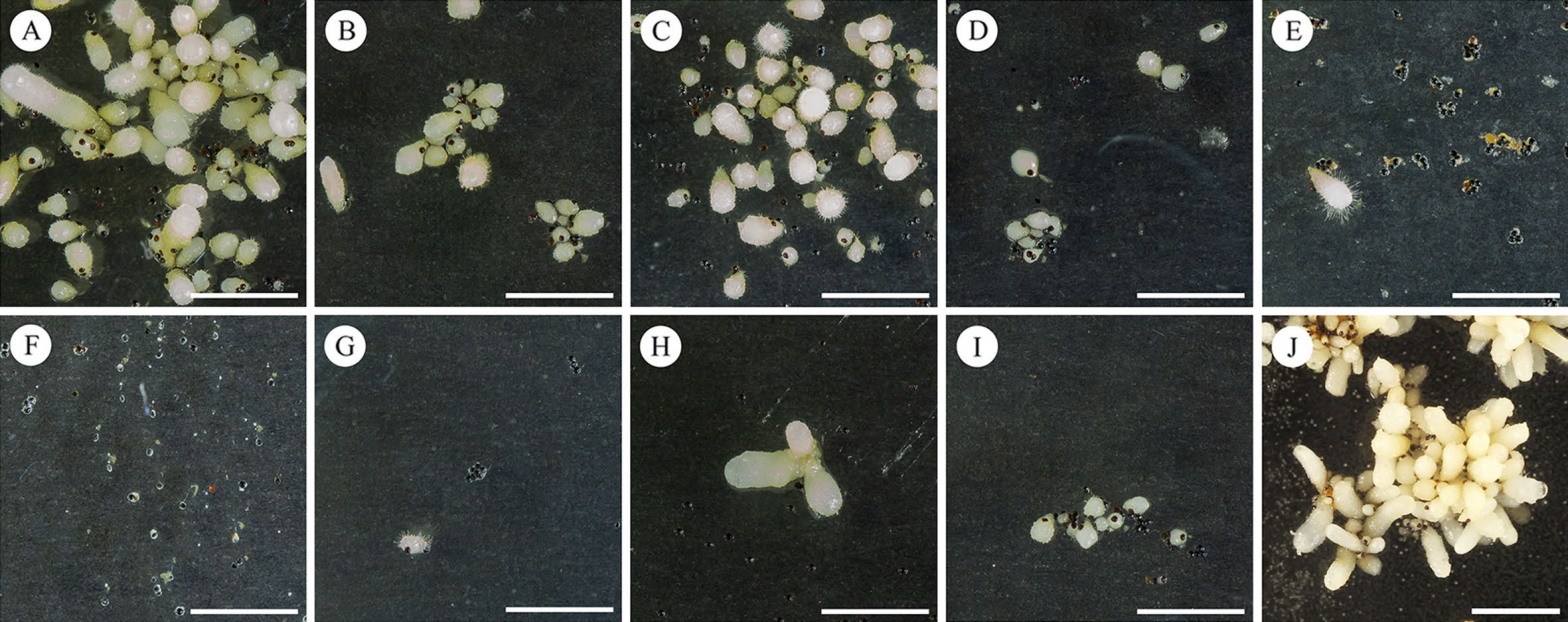
Representative protocorms of *Vanilla planifolia* after different treatments of seeds. Cultures were incubated for 5 months on BM1 medium at 30 °C in dark. Mature seeds were disinfected with solutions of HCl (A–D), solutions of H_2_SO_4_ (**E**—Immature seeds excerpted from surface sterilized unripe pods served as a positive control as they lack dormancy (**J**). The seed treatments were (in the same order as in Fig. 1A): 30 min 0.1 M HCl (A), 30 min 0.1 M HCl + pepsin 0.5 g/100 ml (**B**), 4 h 0.1 M HCl + pepsin 0.5 g/100 ml (**C**), 15 min 35–38% HCl (**D**), 3 min 50% H_2_SO_4_ (**E**), washed in 50% H_2_SO_4_ (**F**), 5 min 50% H_2_SO_4_ (**G**), 5 min 96% H_2_SO_4_ (**H**), 30 min Chlorinated lime 66 g/l (**I**). Scale bars 1 cm

Protocorm growth was significantly affected by disinfection treatments [F _(5,1834)_ = 63.61, P < 2 × 10^–16^] and the differences were similar as in the case of seed germination (Figs. 1B; 2, Additional file 2: Table S1). Immature seeds grew into significantly larger protocorms than mature seeds regardless the treatment (Fig. 1B, Additional file 3: Figure S2). However, the difference was not as pronounced as in the case of germination. Also, germination percentage of immature seeds varied very little but growth of protocorms from immature seeds varied more than the growth of protocorms from mature seeds (with exception of mature seeds treated with 36–38% HCl for 15 min). Protocorms from mature seeds treated with hydrochloric acid grew significantly less than the protocorms from immature seeds, but there was no significant difference in growth in regard of HCl concentration or presence of pepsin applied to mature seeds. Calcium hypochlorite treatment of mature seeds led to significantly smallest growth of protocorms among all variants. Sulfuric acid treatments were excluded from testing because only very few protocorms grew from these seeds. However, mature seeds treated with sulfuric acid relatively mildly (incubation in 96% H_2_SO_4_ for 5 min, wash in 50% H_2_SO_4_ and incubation in 50% H_2_SO_4_ for 5 min) grew into few very large protocorms (1–4 per Petri dish). Variants treated with sulphuric acid were omitted from protocorm size comparison (Fig. 1B), because only a very limited number of protocorms grew after these treatments. However, some of those protocorms were large (mean value 2.9 ± 1,5 mm; Fig. 2), showing that sulphuric acid treatment can probably break seed dormancy, but the treatment used was too strong and killed most of the seeds and probably only the fittest survived.

## Discussion

We received much higher germination percentages than previously reported in asymbiotic in vitro cultures of any *Vanilla* species. The main reason seems to be a novel disinfection/scarification of seeds because the germination percentage after conventional disinfection does not differ too much from those previously reported. However, our cultivation protocol differs from all others in several points which could likely improve the germination percentage in concert. We used cultivation medium BM-1 which works very well with a variety of difficult-to-germinate terrestrial orchid species (van Waes and Debergh 1986; Rasmussen 1995), for the first time in *Vanilla*. *Vanilla planifolia* is a secondary hemiepiphytic liana with endozoochorous seeds which likely germinates terrestrially, so cultivation requirements more similar to terrestrials than epiphytes could be expected. Comparison of different media could allow other improvement of the efficiency in next studies.

Another of our innovations is cultivation at high temperature of 30 °C. Unfortunately, two previously published papers dealing with asymbiotic germination of some *Vanilla* species in vitro do not specify temperature used for cultivations (Chaipanich et al. 2020; Yeh et al. 2021). Nevertheless, it could be expected that 30 °C was not their choice as such a temperature is very uncommon for in vitro cultivations. Knudson (1950) found that *V. fragrans* germinated the best at the highest temperature tested, 32 °C, with no germination at 25 °C. We compared germination at two temperatures, 25 °C and 30 °C with germination present at the higher temperature only. Thus, it seems likely that high cultivation temperature could be beneficial for germination of *Vanilla* in general.

Because of a hard seed coat of *Vanilla* seeds and the supposition of the existence of physical dormancy (Yeh et al. 2021), we focused mainly on treatment of seeds before sowing, acting simultaneously as a disinfection and chemical scarification (e.g., Rasmussen 1995; Magrini and De Vitis 2017; Katsalirou et al. 2019). We used two main groups of chemicals: (i) hypochlorite solutions which are conventionally used for treating orchid seeds and (ii) acid solutions (some of them with pepsin) which are sometimes used to simulate degradation of non-orchid seeds in digestive tract during endozoochorous seed dispersal. We achieved relatively high germination percentages with specific variants of both approaches. One relatively efficient way was treatment of seeds with strong calcium hypochlorite solution. Higher efficiency of calcium hypochlorite over sodium hypochlorite for induction of seed germination has been reported in various orchid species (e.g., Rasmussen 1995; Vejsadová 2006; Ponert et al. 2011). Thus, it is not surprising that the germination percentage achieved by us (mean value 29.8%) is much higher than previously reported with sodium hypochlorite (12.7%, Yeh et al. 2021) but similar to that previously reported with calcium hypochlorite (27%, Knudson 1950). However, it is also possible that the higher germination percentage achieved in our experiment may be partly caused by the longer cultivation time—5 months compared to 2 months Yeh et al. (2021).

Acidic solutions are generally used to overcome physical part of dormancy in endozoochorous seeds (e.g., Gunes et al. 2013; Vazačová and Münzbergová 2013; Jaganathan et al. 2019). In our experiments, strong sulphuric acid was probably too strong, leading to the disintegration of the seeds and little or no germination. Pansarin (2021) found that a very short treatment with sulphuric acid (60 s, unspecified concentration) can induce germination of various *Vanilla* species including *V. planifolia*. On the other hand, solutions of hydrochloric acid were highly efficient in our experiments, leading to the significantly highest germination percentages (mean value up to 64.1%), irrespective of the strength of the acid and presence of pepsin. These results not only make vanilla sowing significantly more efficient, but also support the hypothesis that vanilla seeds are adapted for endozoochory. It seems that *V. planifolia* seeds are dispersed by birds (Pansarin 2021) nevertheless a role of mammals is also possible as these fruits are highly aromatic (Pansarin and Suetsugu 2022). The HCl solutions are frequently used to mimic conditions in the animal digestive tract and to broke impermeable coats of endozoochorous seeds (e.g., Venier et al. 2012; Kleyheeg et al. 2018; Jaganathan et al. 2019) and it likely acts the same way with our seeds. At this point, it could be proposed that the seeds of *V. planifolia* possess physical dormancy, typical for endozoochorous seeds (Baskin and Baskin 2021; Pansarin 2021). However, the mechanisms regulating germination and dormancy of orchid seeds are poorly understood and based on the available results we cannot clearly conclude what type of dormancy is involved. However, we can discuss what the existing results indicate.

Numerous evidence from various orchid species suggest that the permeability of testa can play a role in some difficult-to-germinate species (e.g., Rasmussen 1995; Lee et al. 2007; Barsberg et al. 2013; Magrini et al. 2019). This is even more likely in *Vanilla* which possess hard seeds adapted to endozoochory (Pansarin 2021; Yeh et al. 2021). Our results indicate that treatment of seeds by corrosive solutions strongly induce germination, likely as a result of testa degradation. Similar results (yet with much lower germination percentages) were obtained with a sodium hypochlorite solution (Yeh et al. 2021) or with a very brief treatment with sulphuric acid (Pansarin 2021). However, available data does not allow identification of exact mechanism responsible for induction of germination, so we may not conclude any specific type of dormancy in these seeds. In some other orchids it has been observed that treatment of seeds with hypochlorite solutions not only increases permeability of testa, but also reduces content of endogenous abscisic acid (Van Waes 1984; Lee et al. 2007), indicating that hypochlorite can affect physiological dormancy. On the other hand, a positive correlation between permeability of testa and germination was observed in orchid *Anacamptis morio* after enzymatic digestion of seed coats (Lindén 1992; Pierce et al. 2019) indicating that hypochlorite can affect also physical dormancy and that the permeation of testa is essential for induction of germination. In our experiments, the HCl solutions simulating digestive tract of animals induce germination even more than the conventionally used alkaline and strongly oxidative hypochlorite solution. Taking into account the adaptation of *Vanilla* seeds for endozoochory (Pansarin 2021; Yeh et al. 2021; Pansarin and Suetsugu 2022), it could likely be expected that the increased permeability of the seed coats is the cause of the increased germination. The dormancy in orchid seeds has been generally classified as morphological or morphophysiological (Baskin and Baskin 2021). In the case if permeability of testa will be confirmed as the causal mechanism of dormancy, the classification would need to be modified to also include the physical composition of dormancy, resulting in ‘‘Class 5. Combinational’’ dormancy (according to the most recent classification of Baskin and Baskin 2021).

## Conclusions

Artificial germination of mature seeds of many orchid species has become routine, but germination of the economically most important orchid, the vanilla, has so far been difficult and unpredictable. Our results have made this technique considerably more efficient and reproducible. The treatment of *Vanilla planifolia* seeds with hydrochloric acid, which is presented in this article, represents the most efficient way for germination of mature seeds of any commercially important species of *Vanilla* reported so far. The high efficacy of this treatment probably reflects the adaptation of vanilla seeds to endozoochory and it’s very likely that the seeds possess a physical component of dormancy. However, further studies are necessary to completely understand the mechanisms of seed dormancy in this species.

## Supplementary Information

**Additional file 1: Figure S1.** Ripe fruits of *Vanilla planifolia* used for sowing of mature seeds. The fruits were collected when they opened their apical part as shown in these pictures, which was approximately 12 months after pollination.

**Additional file 2: Table S1.** Composition of cultivation medium BM1. The pH of medium was adjusted before autoclaving to 5.8 with 1M KOH after final volume was made up with distilled water.

**Additional file 3: Table S2.** Statistics for germination percentage and protocorm size of *V. planifolia* after 5 months of asymbiotic cultivation on BM1 medium at 30 °C in dark. Germination percentage was analysed with Welch’s F-test (F _(9,28)_ = 84.99, P < 2 × 10^-16^) and protocorm size was analysed with Nested ANOVA (F _(5,1834)_ = 63.61, P < 2 × 10^-16^). SEM means the standard error of the mean.

## Data Availability

The datasets used and/or analysed during the current study are available from the corresponding author on reasonable request.
