# Supplementary material for: Acid scarification as a potent treatment for an in vitro germination of mature endozoochorous Vanilla planifolia seeds
Source: Bot Stud. 2023 Apr 17;64:9. doi: 10.1186/s40529-023-00374-z (PMC10110789; doi:10.1186/s40529-023-00374-z)
Supplement: Supplementary file 1 — Additional file 1: Figure S1. Ripe fruits of Vanilla planifolia used for sowing of mature seeds. The fruits were collected when they opened their apical part as shown in these pictures, which was approximately 12 months after pollination. [file 40529_2023_374_MOESM1_ESM.docx]

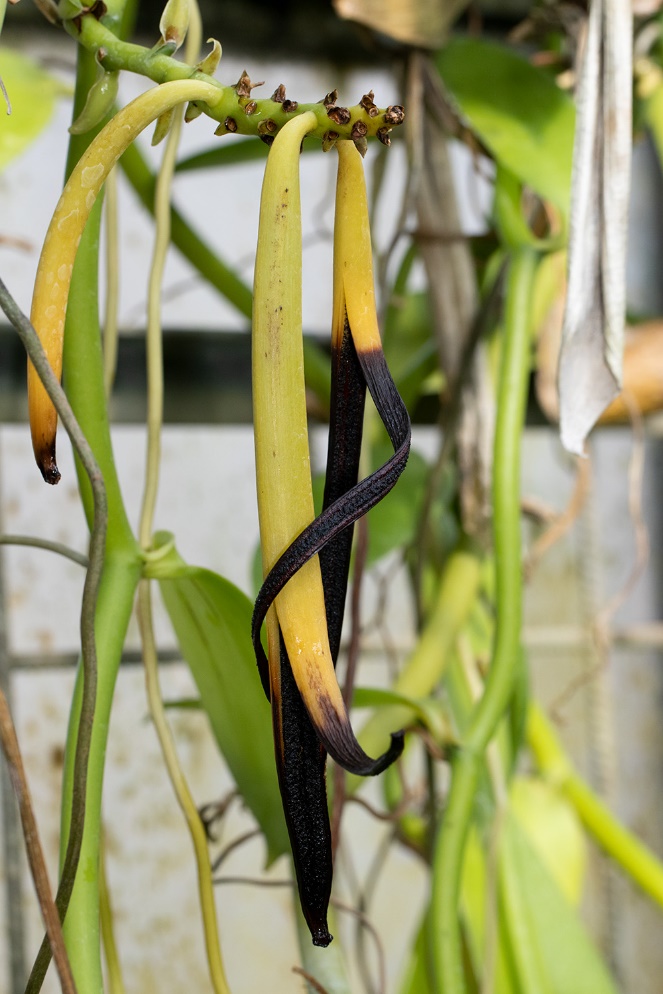

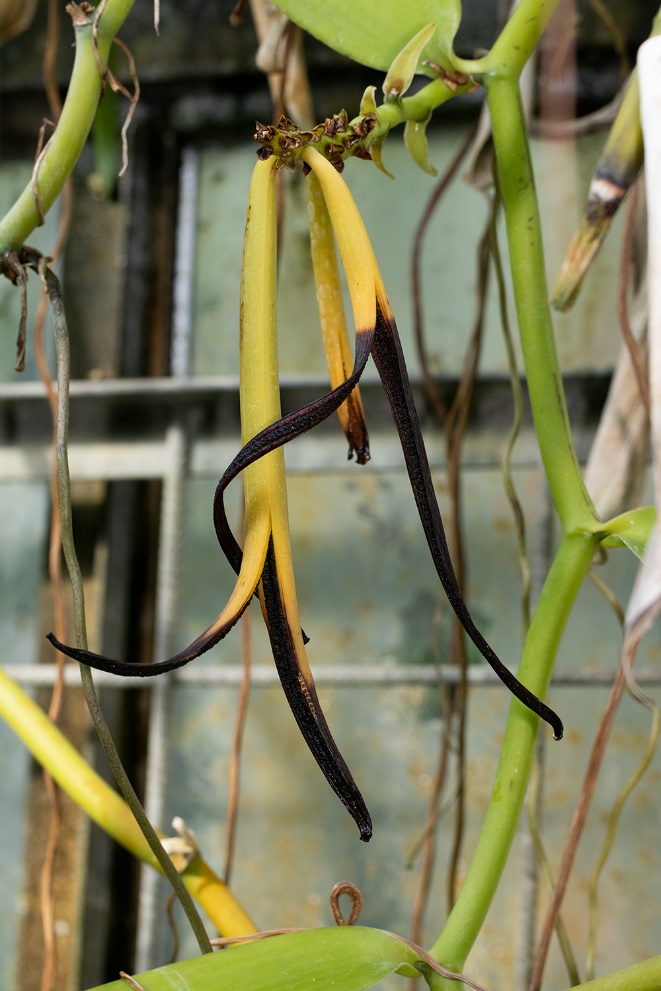


## Additional file 1: Figure S1

Ripe fruits of *Vanilla planifolia* used for sowing of mature seeds. The fruits were collected when they opened their apical part as shown in these pictures, which was approximately 12 months after pollination.
