# Supplementary material for: Acid scarification as a potent treatment for an in vitro germination of mature endozoochorous Vanilla planifolia seeds
Source: Bot Stud. 2023 Apr 17;64:9. doi: 10.1186/s40529-023-00374-z (PMC10110789; doi:10.1186/s40529-023-00374-z)
Supplement: Supplementary file 2 — Additional file 2: Table S1. Composition of cultivation medium BM1. The pH of medium was adjusted before autoclaving to 5.8 with 1M KOH after final volume was made up with distilled water. [file 40529_2023_374_MOESM2_ESM.docx]

## Additional file 2: Table S1

| **Ingredients** | **Amount (mg/l)** |
| --- | --- |
| Magnesium sulphate | 100.000 |
| Potassium phosphate monobasic | 300.000 |
| Boric acid | 10.000 |
| Cobalt chloride hexahydrate | 0.025 |
| Copper sulphate pentahydrate | 0.025 |
| EDTA disodium salt dihydrate | 37.250 |
| Ferrous sulphate heptahydrate | 27.850 |
| Manganese sulphate monohydrate | 25.000 |
| Molybdic acid (sodium salt) | 0.213 |
| Zinc sulphate heptahydrate | 10.000 |
| Biotin | 0.050 |
| Folic acid | 0.500 |
| Myo-Inositol | 100.000 |
| Nicotin acid (free acid) | 5.000 |
| Pyridoxine HCl | 0.500 |
| Thiamine hydrochloride | 0.500 |
| Glycine | 2.000 |
| L-Glutamine | 100.000 |
| Sucrose | 20000.000 |
| Casein hydrolysate | 21.200 |
| CleriGel | 3000.000 |

Composition of BM1 medium. The pH of medium was adjusted before autoclaving to 5.8 with 1M KOH after final volume was made up with distilled water.
