# Supplementary material for: Acid scarification as a potent treatment for an in vitro germination of mature endozoochorous Vanilla planifolia seeds
Source: Bot Stud. 2023 Apr 17;64:9. doi: 10.1186/s40529-023-00374-z (PMC10110789; doi:10.1186/s40529-023-00374-z)
Supplement: Supplementary file 3 — Additional file 3: Table S2. Statistics for germination percentage and protocorm size of V. planifolia after 5 months of asymbiotic cultivation on BM1 medium at 30 °C in dark. Germination percentage was analysed with Welch’s F-test (F (9,28) = 84.99, P < 2 × 10^-16^) and protocorm size was analysed with Nested ANOVA (F (5,1834) = 63.61, P < 2 × 10^-16^). SEM means the standard error of the mean. [file 40529_2023_374_MOESM3_ESM.docx]

## Additional file 3: Table S2

|  | **germination rate** | | | | | **protocorm size** | | |
| --- | --- | --- | --- | --- | --- | --- | --- | --- |
| **treatment** | **mean (%)** | **SEM (%)** | **number of  plates** | **seeds per plate** | | **mean (µm)** | **SEM (µm)** | **number of protocorms measured** |
|  |  |  |  | **mean** | **SEM** |  |  |  |
| 5 min 96% H_2_SO_4_ | 3.29 | 0.49 | 4 | 106.75 | 1.75 | - | - | - |
| 15 min 96% H_2_SO_4_ | 0 | - | 4 | 102.24 | 3.03 | - | - | - |
| 30 min 96% H_2_SO_4_ | 0 | - | 4 | 109.05 | 4.97 | - | - | - |
| 30 min  0.1 mol HCl + pepsin + Tween 20 | 60.92 | 6.03 | 4 | 113.00 | 11.29 | 1228.49 | 31.53 | 329 |
| 30 min 0.1 mol HCl | 60.04 | 4.16 | 4 | 135.50 | 11.89 | 1474.74 | 28.03 | 400 |
| 4 h 0.1 mol HCl + pepsin + Tween 20 | 49.66 | 4.92 | 4 | 137.25 | 25.27 | 1325.76 | 29.77 | 318 |
| 15 min 35-38% HCl | 64.31 | 10.51 | 4 | 61.67 | 3.67 | 1513.15 | 82.96 | 98 |
| 3 min  50% H_2_SO_4_ | 4.00 | 0.72 | 4 | 114.75 | 4.92 | - | - | - |
| washed in 50% H_2_SO_4_ | 2.91 | 0.26 | 4 | 102.67 | 15.84 | - | - | - |
| 5 min 50% H_2_SO_4_ | 2.85 | 0.89 | 4 | 95.25 | 12.77 | - | - | - |
| 30 min  66g/l Ca(ClO)_2_ | 29.79 | 4.91 | 4 | 155.00 | 13.64 | 938.05 | 25.23 | 276 |
| unripe pods sterilized with 200g/l Ca(ClO)_2_ | 96.68 | 0.24 | 4 | 105.25 | 1.93 | 1773.55 | 43.55 | 400 |

Statistics for germination percentage and protocorm size of *V. planifolia* after 5 months of asymbiotic cultivation on BM1 medium at 30 °C in dark. Germination percentage was analysed with Welch’s F-test (F _(9,28)_ = 84.99, P < 2 × 10^-16^) and protocorm size was analysed with Nested ANOVA (F _(5,1834)_ = 63.61, P < 2 × 10^-16^). SEM means the standard error of the mean.
